# The Attenuated Pseudorabies Virus Vaccine Strain Bartha K61: A Brief Review on the Knowledge Gathered during 60 Years of Research

**DOI:** 10.3390/pathogens9110897

**Published:** 2020-10-27

**Authors:** Jonas L. Delva, Hans J. Nauwynck, Thomas C. Mettenleiter, Herman W. Favoreel

**Affiliations:** 1 Department of Virology, Parasitology and Immunology, Faculty of Veterinary Medicine, Ghent University, Salisburylaan 133, 9820 Merelbeke, Belgium; jonas.delva@ugent.be (J.L.D.); hans.nauwynck@ugent.be (H.J.N.); 2 Friedrich-Loeffler-Institut, Federal Research Institute for Animal Health, Südufer 10, 17493 Greifswald-Insel Riems, Germany

**Keywords:** Suid herpesvirus 1, Aujeszky’s disease virus, pseudorabies virus, modified live vaccine, Bartha, Bartha K61

## Abstract

Pseudorabies virus (PRV) is a member of the alphaherpesvirus subfamily of the herpesviruses and is the causative agent of Aujeszky’s disease in pigs, causing respiratory, neurological, and reproductive symptoms. Given the heavy economic losses associated with Aujeszky’s disease epidemics, great efforts were made to develop efficacious vaccines. One of the best modified live vaccines to this day is the attenuated Bartha K61 strain. The use of this vaccine in extensive vaccination programs worldwide has assisted considerably in the eradication of PRV from the domesticated pig population in numerous countries. The Bartha K61 strain was described in 1961 by Adorján Bartha in Budapest and was obtained by serial passaging in different cell cultures. Ever since, it has been intensively studied by several research groups, for example, to explore its efficacy as a vaccine strain, to molecularly and mechanistically explain its attenuation, and to use it as a retrograde neuronal tracer and as a vector vaccine. Given that the Bartha K61 vaccine strain celebrates its 60th birthday in 2021 with no sign of retirement, this review provides a short summary of the knowledge on its origin, characteristics, and use as a molecular tool and as a vaccine.

## 1. Introduction

Herpesviruses are a large family of double-stranded DNA (dsDNA) viruses containing pathogens of man and animals. The family of herpesviruses is subdivided into three subfamilies: alpha-, beta-, and gamma-herpesviruses. The alphaherpesviruses represent the largest subfamily of the herpesviruses. There are three alphaherpesviruses with man as their natural host: herpes simplex virus 1 (HSV-1), herpes simplex virus 2 (HSV-2), and varicella zoster virus (VZV). Besides man, numerous animals serve as natural hosts for specific alphaherpesvirus species [1]. Examples include bovine herpesvirus 1 in cattle [2], feline herpesvirus 1 in cats [3], equine herpesvirus 1 in horses [4], and pseudorabies virus (PRV) in pigs [5].

PRV or Suid alphaherpesvirus 1 is the causative agent of Aujeszky’s disease (AD) in swine, causing respiratory, neurological, and reproductive symptoms. The infection of PRV in pigs typically starts in the nasal mucosa, after which viral particles infect sensory neuronal cells. Through retrograde neuronal transport, viral particles travel toward the trigeminal ganglia and olfactory bulb [6,7,8,9], where the virus enters a latent stage of infection [10]. Reactivation and renewed production of infectious particles are infrequent in PRV, but can be induced by external factors such as stress from transport or handling. Subsequently, via anterograde transport, virions may reach the mucosa and reestablish local lytic replication cycles. Viral particles can then be shed and infect new pigs [11].

Besides pigs, there is a very broad range of mammals that are susceptible to PRV infection, such as cattle, sheep, rabbits, cats, dogs, guinea pigs, rats, and mice [12]. However, pigs are the only susceptible animals that are able to survive AD, although disease prognosis is highly dependent on factors such as inoculation site, viral strain and titer, and age of pigs. In particular, young piglets develop neurological symptoms with high mortalities up to 100% for neonatal piglets, while older animals suffer respiratory symptoms, fever, general dullness, and reduced appetite [13]. In pregnant sows, AD may cause abortion or stillbirth [14]. Consequently, PRV epidemics have led to extensive economic losses in the pork production industry [15].

## 2. History of Aujesky’s Disease Vaccines

In the second half of the 20th century, the farming landscape in the western world became more industrialized. For pig farms, this included substantial changes in animal husbandry, confinement of larger numbers of pigs, and continuous farrowing. Concurrently, outbreaks of Aujeszky’s disease (AD) occurred more frequently and were more severe. As a result of the severe economic losses, substantial efforts were made to control the disease [13]. Elaborate and intensive disease control programs were implemented based on disinfection, outbreak monitoring, removal of PRV affected animals, and movement and trade restrictions. Nevertheless, it was not until vaccination was employed and made mandatory that full eradication was possible [16]. As a result of these thorough eradication programs, many European countries, the USA, Mexico, Canada, and New Zealand achieved an AD-free status [17]. As soon as a country successfully achieved an AD-free status, vaccination was commonly prohibited [17]. It should be mentioned, however, that PRV remains endemic in the wild boar population, hence care has to be taken to prevent spillover into the domesticated pig population [18]. Although many countries reached and maintained an AD-free status, new and antigenically different virus strains emerged in northern China at the end of 2011, causing severe PRV outbreaks, including in vaccinated swine farms [19].

The first-generation vaccines against AD consisted of both inactivated and live attenuated PRV. However, it was readily observed that live attenuated vaccines induced better antiviral responses, especially when administered at a high titer and combined with an adjuvant [20,21]. These attenuated vaccines were usually made by extensive serial passaging of the virus on (non-)natural host cell cultures. The first live vaccines to be produced by serial passage were the Bartha and BUK strains [22,23]. A molecular analysis of these strains revealed a partial deletion of the unique short (US) region containing the gene encoding the viral glycoprotein gE, a major virulence factor [24]. These insights, together with new advances in molecular biology, led to a new generation of rationally designed vaccines carrying single or multiple deletions in non-essential virulence genes, such as gE and the viral thymidine kinase (TK) [25,26]. The use of these so-called marker vaccines allows to differentiate infected from vaccinated animals. Only infected animals elicit a strong antibody response toward the gE glycoprotein, while both vaccinated and infected animals elicit antibodies toward other viral proteins, such as the gB glycoprotein. This allows for the development of serological assays (e.g., ELISA) that can discriminate between infected, vaccinated, and naive animals. This method is called the DIVA (Differentiating Infected from VAccinated animals) strategy and has been instrumental in pathogen eradication programs around the world [27].

## 3. History of the Bartha K61 Strain

**Figure 1:**
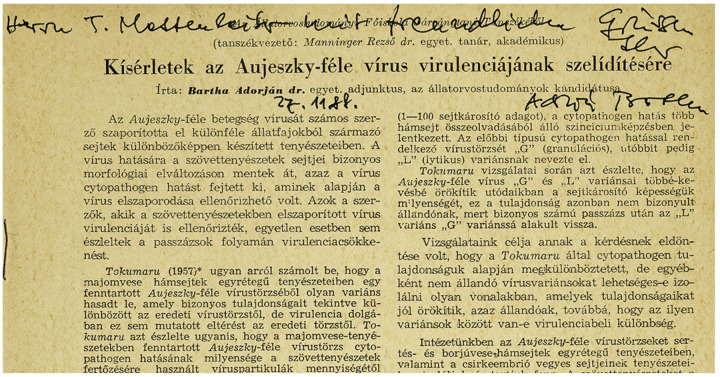
Personalized first page of the 1961 paper by Prof. Dr. Adorján Bartha on the development of the pseudorabies virus (PRV) Bartha vaccine strain, directed at *Mr. T. Mettenleiter* ‘with kind regards/mit freundlichen Grüßen’, dated 27 November 1988.

One of the first attenuated PRV vaccines ever developed is the Bartha K61 strain, which remains a golden standard vaccine in various AD eradication programs to this day. It was first reported in 1961 by Adorján Bartha, a Hungarian veterinarian [22] (Figure 1). He described a less virulent variant of a field strain obtained by serial passaging. The latter process consists of repeatedly propagating a virus on specific cell cultures, usually non-natural host cells. Consequently, genomic mutations can accumulate in a virus progeny. Often, there is a positive selection toward mutations that allow for better viral replication in this particular cell culture. Yet, some of these acquired mutations might actually impede infection in the natural host. Hence, after numerous passages, a new mutant strain might be obtained that is attenuated in the natural host because of an altered expression or even complete deletion of genes encoding certain virulence or immune-evasion factors.

For the Bartha K61 strain, it was not described how many passages were performed exactly or on which cells specifically. However, the manuscript did mention that the PRV strains at the institute were maintained on calf and pig kidney epithelial cells and mixed chicken embryo cells [22]. The variant strain obtained after passaging was subjected to further analyses and it was observed that it formed very small plaques and no syncytia on porcine kidney cell monolayers, in contrast to the virulent strain. The variant was also phenotypically stable and did not produce symptoms of disease in several domesticated animals, indicating it had reduced virulence. During the following years, experimental work confirmed its safety, efficacy, and effectiveness as a vaccine for AD [28,29,30]. In fact, when it was compared with other attenuated vaccine strains, it was shown to be highly efficacious in challenge assays when body weight gain, viral shedding, and fever post-challenge were compared [20,31]. The attenuated variant strain became eventually known as Bartha K61, named after the small plaque phenotype (small is translated to “kicsi” in Hungarian, thus the abbreviation K) observed in 1961, hence K61 [22]. In Europe, Bartha K61 played a central role in PRV eradication, as it was the vaccine employed in many successful eradication programs [17].

Nevertheless, despite the success of the Bartha K61 vaccine in Europe, recently new virulent PRV strains emerged in Bartha K61-vaccinated pig farms in China [32]. Experimental inoculations showed that some of these strains indeed may be more virulent in unvaccinated pigs [33]. However, whether the Bartha K61 vaccine provided insufficient protection is still under debate, as crucial information is missing on how vaccination programs were performed as these are not mandatory in China. Moreover, in-depth information about the quality of locally licensed and produced PRV vaccines is not always readily available [34]. In addition, experimental assays suggesting incomplete protection provided by Bartha K61 against the novel Chinese PRV strains were often performed in mice or sheep, susceptible, but non-natural, hosts that are difficult to extrapolate to pigs [32,33,35]. Several reports show that Bartha K61-vaccinated animals in fact are still clinically protected against lethal challenges by several recent, highly virulent, Chinese strains including XJ5 and AH02LA [36,37,38]. A recent publication however reported mortality of two out of five Bartha-vaccinated pigs when challenged with the highly virulent strain HB1201 [39]. There appears to be a growing consensus that Bartha K61 may show reduced efficacy toward some recent Chinese PRV strains due to antigenic differences of these new strains, including, those in the immunogenic viral gC and gD glycoproteins [39]. More in-depth immunological research is required to fully understand the antigenic differences that may affect the protective effects of Bartha K61 against different PRV field strains.

## 4. Genetic Background of the Bartha K61 Strain

As the attenuated Bartha strain was obtained by serial passaging, it was initially not known which changes were introduced in the viral genome. Hence, efforts were made to explain its safety and efficacy as a live attenuated vaccine. A first important discovery by Lomniczi and colleagues in 1984 showed that Bartha K61 contains a large deletion in the US portion of the genome [40], resulting in full deletion of the genes encoding gE and US9 and a partial deletion of the genes encoding gI and US2 [24,41]. Interestingly, it was later revealed that there is a strong selection toward gE deletion when PRV is cultivated and passaged in chicken embryo fibroblasts, one of the cell types the Bartha K61 strain was presumably passaged on [42]. The gE glycoprotein forms a heterodimer with the gI glycoprotein [43]. Together they are involved in virulence [44] and are required for anterograde neuronal transport of viral particles [45]. Similarly, pUS9 is also involved in anterograde neuronal spread of viral particles via sorting of virus particles toward the axon termini [46]. Consequently, anterograde, but not retrograde, neuronal transport of Bartha K61 virions is abolished [47]. This means that post-vaccination, Bartha K61 virions can reach the peripheral nervous system via retrograde transport (though less efficiently compared with wild-type PRV [48]), resulting in the induction of latency. However, if reactivation occurs, newly formed Bartha virions are not excreted because of the abolished anterograde transport. In addition, the deletion of gE also plays a role in the small plaque phenotype observed in different cell cultures [49,50]. It is assumed that the gE/gI heterodimer complex guides viral particles toward cell junctions, thereby stimulating cell-to-cell spread and plaque formation [51].

On the other hand, pUS2 has no function in neuronal spread [52]. It has been reported to modulate ERK (extracellular signal-regulated kinases) signaling by direct sequestering of ERK1/2 and hence, preventing the activation of its nuclear targets [53]. Moreover, US2 deletion mutants of PRV have been reported recently to show higher viral titers early in infection in porcine cerebral cortex primary cells [54]. Whether this has an influence on the performance of the Bartha vaccine is unknown.

Whereas the experiments of Lomniczi validated that the genes encoded in the US region play a role in PRV virulence, repair of this region did not restore virulence of the Bartha strain, suggesting additional virulence factor(s) outside the US region to be involved [55]. Klupp and colleagues identified this additional virulence factor as UL21 [56]. Indeed, when both the US region and UL21 were repaired to wild-type PRV sequences, the rescue mutant regained virulence for pigs and 1-day-old chickens, although not to wild-type levels [57]. In contrast to the US mutation which constitutes a large deletion, the UL21 gene in Bartha K61 contains mutations resulting in only three amino acid substitutions compared with wild-type PRV [56]. Subsequent research reported that these mutations impaired efficient retrograde transneuronal spread [58]. The mutations in Bartha UL21 affect the incorporation of tegument proteins as Bartha virions contain significantly reduced or nearly undetectable amounts of pUS3, pUL46, and pUL49, whereas repair of the Bartha UL21 locus restored virion incorporation of these tegument proteins [59,60]. Although pUL21 of PRV has been recently reported to interact with the cellular trafficking protein Roadblock-1 [61], it is unclear if this interaction is impaired in Bartha K61.

Besides the US and UL21 mutations, additional mutations that may be of significance have been detected. gC is a structural glycoprotein involved in attachment of PRV virions to the host cell and plays a role in virulence [62,63]. The signal sequence of Bartha gC is mutated at leucine 14 to a proline, causing less efficient sorting, glycosylation, and subsequent incorporation in the viral particle [64]. Importantly, when the gC sequence was repaired in the partially virulent Bartha K61 rescued for the US deletion and UL21, virulence in 1-day-old chickens was similar to wild-type levels [62]. Additionally, Bartha K61 was found to be poorly released from RK-13, but not PK-15, cells [49]. Similar to the small plaque phenotype, the deletion of gE partially explains this reduced virus release [44]. However, when the gC gene was repaired, virions were released from RK-13 cells to near wild-type amounts, independently of gE repair [63]. gM is also a structural viral glycoprotein that has one single *N*-glycosylation site in wild-type PRV. The amino acid sequence of Bartha K61 gM shows an alteration in the consensus *N*-glycosylation signal, resulting in a loss of *N*-glycosylation. Only small phenotypic alterations such as slightly delayed entry or reduced plaque size were associated with this mutation [65].

Our lab recently performed proteomic analyses on Bartha K61 and several wild-type PRV virions. We found that Bartha K61 virions, in contrast to wild-type PRV virions, incorporate almost no IE180 protein (Delva and Favoreel, unpublished observations), a viral transactivator protein that is expressed with immediate early kinetics in infected cells. This viral protein is in fact the sole immediate early protein of PRV and functions as an important transcription factor regulating viral and even cellular gene transcription [66]. Whether this reduced virion incorporation influences virus–cell interactions, is currently unknown.

In 2011, the complete genome sequence of Bartha K61 was reported and compared with two wild-type strains, Becker and Kaplan [67]. This study detected an additional 46 proteins with coding differences unique to Bartha K61 and not found in either wild-type strain. Some of them were minor, e.g., A13V in VP18.8 (UL13), and others occurred in non-conserved regions with high inter-strain variability as was the case for genes encoding VP1/2 (UL36) or IE180. However, other alterations were detected in functional domains of key proteins such as the viral glycoproteins gB, gH, and gN. Both gB and gH are involved in membrane fusion during virus entry into a host cell [68]. Alphaherpesvirus fusion is executed by the joint action of gD, gH, gL, and gB. The gH protein of Bartha contains a P438S mutation, which alters the tertiary structure of the protein, as this proline induces a bend in an alpha-helix, aiding a highly conserved disulfide bond in the gH core [69]. The introduction of this mutation into the gH gene of the wild-type PRV strain Kaplan resulted in the formation of small plaques and delayed penetration kinetics [70]. Hence, although gE was shown to partially contribute to the small plaque phenotype of Bartha K61, this P438S mutation in gH is likely also involved. Additional research is necessary to confirm this, for example, by analyzing the effect of repair of this P438S mutation. Furthermore, Bartha K61 gB harbors three unique mutations immediately near its furin cleavage site, i.e., S506A, P507A, and P509Q. This cleavage has been shown to affect in vitro cell–cell spread of PRV and replication of VZV in human skin xenografts [71,72]. Nevertheless, it was confirmed that Bartha K61 gB is still cleaved in PK-15 cell cultures [59,67], but it is unknown if the cleavage efficiency or any alternative functions of gB are altered, such as immunomodulation [73]. Lastly, gN of Bartha has a L7P substitution in the signal sequence, perhaps altering glycoprotein processing and packaging, as was observed for gC [64].

## 5. The Bartha K61 Strain as a Neuronal Tracer

Besides the use of Bartha K61 as an attenuated vaccine, it has also been widely used as a retrograde neuronal tracer [74]. As mentioned above, Bartha K61 particles can no longer perform anterograde spread because of the US deletion [47]. This allows researchers to map specific retrograde neuronal connections in the nervous system. Additionally, Bartha-infected non-natural hosts, such as mice, take longer to succumb because of the attenuated phenotype compared with wild-type infected hosts. Consequently, trans-synaptic viral infections are more extensive because Bartha K61 is allowed more time to spread [75]. Examples of retrograde neural mapping using Bartha K61 include the description of interconnected parallel circuits between the nucleus accumbens and the thalamus [76] and new insights in the sleep and waking regulation (circadian rhythm) of the suprachiasmatic nucleus in rats [77].

## 6. The Bartha K61 Strain as a Vector Vaccine

Because of its safe and efficacious profile, the Bartha K61 vaccine has also been implemented as a vector for vaccination against more distantly related PRV strains or even other pathogens. For example, in an effort to efficiently vaccinate against the recently emerged and antigenically different PRV strains in China, research groups have tried to optimize the Bartha K61 vaccine by exchanging several of its immunogenic viral antigens (such as gB, gD, and gC) with those of the emerging Chinese strains [39]. Based on neutralization assays and small-scale vaccination experiments, these strategies seem justifiable, though more in-depth vaccination experiments are necessary to validate their usefulness [35,37]. On the other hand, by introducing foreign antigens derived from other pathogens into the Bartha K61 backbone and subsequent vaccination, it is possible to establish clinical protection against these other pathogens [78]. For example, introduction of the porcine reproductive and respiratory syndrome virus (PRRSV) GP5 gene into the Bartha backbone and subsequent vaccination resulted in significant clinical protection and reduced pathological lesions following PRRSV challenge in piglets [79]. Moreover, vaccination with a Bartha K61 vector expressing the swine influenza H3N2 hemagglutinin induced protection in mice against a virulent challenge of swine H3N2 influenza virus [80]. Similarly, vaccination of pigs with a Bartha vector expressing the H1 hemagglutinin or N1 neuraminidase of the 2009 pandemic H1N1 swine-origin influenza virus significantly inhibited virus replication after challenge with this H1N1 virus [81].

## 7. Immunogenicity of the Bartha K61 Strain

Despite the widespread use of the Bartha K61 strain, studies regarding its potent immunogenicity are relatively scarce. Pol and colleagues observed an influx of mainly lymphocytes and some macrophages and neutrophils in Bartha K61-infected nasal mucosa in pigs, while the wild-type PRV strain NIA-3 and another vaccine strain (2.4N3A) caused an infiltration of primarily neutrophils and macrophages, respectively [82]. Furthermore, previous work at our lab by Lamote and colleagues revealed that compared with different wild-type PRV strains, Bartha K61 induces massively increased type I interferon production in primary porcine plasmacytoid dendritic cells (pDC), a specific subset of dendritic cells specialized in the production of type I interferons [83]. Hence, it appears that Bartha K61 infection causes an innate immune response in pigs that is different and may be more pronounced than that observed with wild-type or even other attenuated vaccine strains of PRV.

Intranasal vaccination of 10-week-old pigs with either live or inactivated Bartha K61 was associated with detection of neutralizing IgG1, IgG2, and IgM antibodies in the serum and IgA antibodies in mucosal fluids (i.e., saliva, tears, and nasal wash), although the responses elicited in animals inoculated with the live vaccine were superior [84,85,86]. Moreover, peripheral blood mononuclear cells (PBMCs) of live Bartha-vaccinated animals, stimulated in vitro with wild-type NIA-3, produced elevated levels of interferon γ (IFNγ) compared with unvaccinated controls and animals vaccinated with inactive Bartha K61, which could be linked to the level of clinical protection post-challenge with wild-type NIA-3 [85]. Reduced or absent virus shedding post-challenge in live Bartha K61-vaccinated pigs also correlated with high frequencies of memory T helper cells and major histocompatibility complex (MHC) class I restricted cytotoxic T lymphocytes [85]. These results suggest that both antibody- and cell-mediated responses are required for an adequate immune response against PRV, and that both are elicited by the live attenuated Bartha K61 vaccine.

Besides these experiments in the natural host, some studies reported on the immune response of Bartha K61 infections compared to wild-type PRV infections in non-natural hosts, particularly in mice. It has been shown that the allele KK of MHC class I molecules is differentially expressed in Bartha K61-infected murine cells compared with cells infected by the wild-type Becker strain. MHC class I molecules present viral antigens and are recognized by cytotoxic T lymphocytes and natural killer (NK) cells. Hence, differential expression of MHC class I alleles might alter immune recognition of these infected cells [87]. Using both mice and rats, it was observed that neuronal infection of Bartha K61 induces a rapid recruitment of microglial cells to infected brain lesions, as part of the host response to the infection [88,89]. Moreover, a transcriptional analysis of Bartha K61-infected brain tissue in rats revealed very strong upregulation of interferon-induced and inflammatory gene transcription close to their death. However, a direct comparison with rats infected with Becker wild-type or Becker deleted for gE/gI was difficult, as these rats succumbed at time points earlier than the time point at which upregulation of interferon/inflammatory responses in Bartha K61-infected rats could be observed [90]. Nonetheless, additional research in rat embryonic fibroblast cells unveiled that wild-type PRV Becker interferes with type I IFN-induced STAT1 phosphorylation, resulting in reduced sensitivity to the antiviral effects of type I interferon. Bartha K61, on the other hand, only partially inhibited type I IFN-induced STAT1 phosphorylation, indicating a greater sensitivity for type I interferons and the concomitant innate immune response [91]. Similarly, a proteomic analysis of brain tissue or synaptosomic tissue from mice infected with Bartha K61 or Becker wild-type revealed several differences in upregulated proteins, which are involved not only in the immune response, but also in cargo transport, signal transduction, cytoskeleton and synapse organization, and metabolic processes [92]. Recently, it has been observed that wild-type Becker, but not Bartha K61, elicits a lethal systemic inflammatory response characterized by high levels of IL-6 and G-CSF in both tissue and serum of footpad-infected mice [93]. Additionally, Bartha K61, but not wild-type Becker, was found to elicit a strong type I IFN response in the footpad and dorsal root ganglion (DRG) neurons of infected mice, without apparent subsequent inflammation in these infected tissues [94,95]. These studies are in line with in vitro studies using porcine cells and porcine pDC [83] illustrating the ability of Bartha K61 to trigger increased type I IFN production compared to wild-type PRV.

In conclusion, it is becoming increasingly obvious that there is more to the Bartha K61 strain than merely a marked reduction in virulence. Multiple studies show that the immune response which is elicited by the Bartha K61 vaccine strain differs functionally from that of several wild-type PRV strains. In addition, some reports indicate that the innate immune response, and more specifically the type I IFN response, plays a central role. As some studies have already established that the deletion of gE/gI is not sufficient to explain the altered immunological responses [83,90], it appears that Bartha K61 has additional immunomodulating tricks up its sleeve. Therefore, it would be interesting to investigate the immunological role of other uniquely altered proteins, such as gC, pUL21, gM, and IE180. Indeed, unraveling the mechanisms underlying the immunogenicity and efficacy of successful vaccines, such as Bartha K61, may potentially aid the design of vaccines against other alphaherpesviruses, like EHV-1 or HSV.

## 8. Concluding Remarks

**Figure 2:**
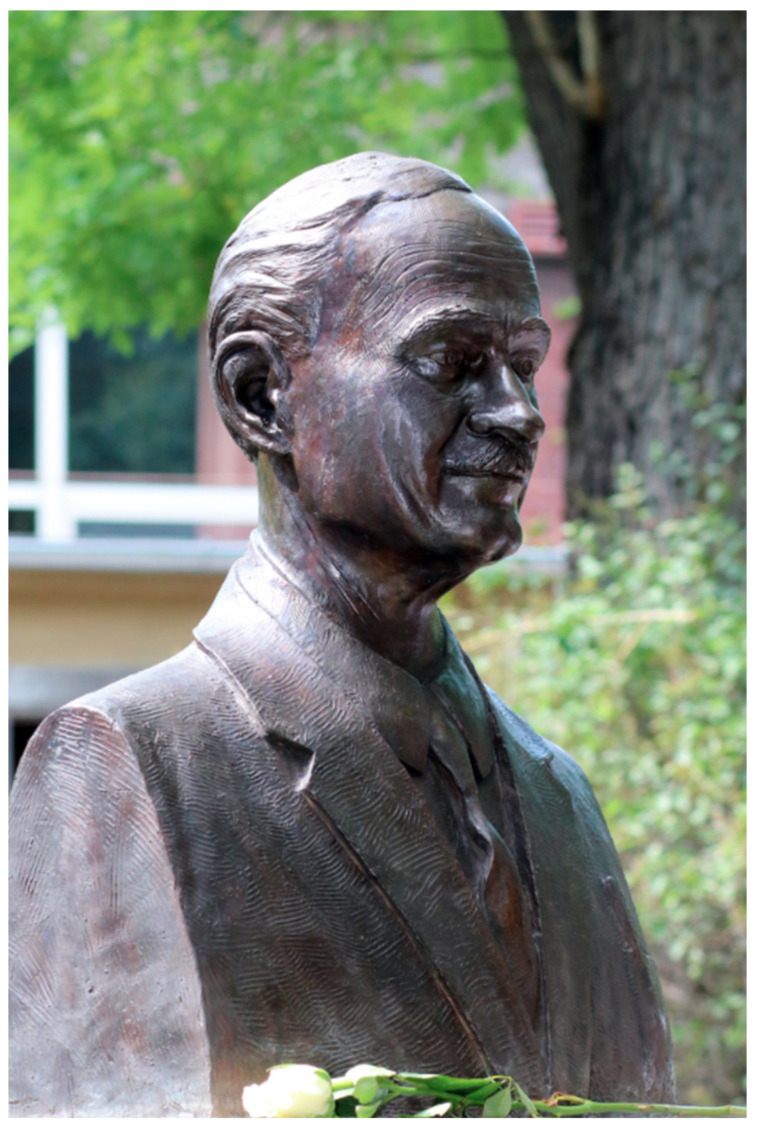
Statue of Prof. Dr. Adorján Bartha in the Park of the University of Veterinary Medicine of Budapest, Hungary. The picture was taken during the festive unveiling of the statue on 24 June 2020 by Gustáv Balázs. Reprinted with permission.

Vaccination has aided PRV eradication from the domesticated pig population to a great extent. The attenuated Bartha K61 strain has proven to be an exemplary vaccine that is both safe and highly efficacious. Based on the construction of this globally used and highly successful vaccine and his many other contributions to science, a statue of Adorján Bartha has recently been installed at his alma mater, the University of Veterinary Medicine in Budapest, Hungary (Figure 2).

Thorough research has identified the large deletion in the US region together with mutations in the UL21- and gC-coding sequences in the Bartha strain as the main factors for its attenuated phenotype and thus safety. Nevertheless, less is known about the reason for its efficacy. Lymphoproliferation post-vaccination with PRV and wild-type challenge has been described [21], as well as NK cell activation [96,97]. However, gaps remain about the interaction of PRV with other immune cells such as dendritic cells. Yet, these cells play a pivotal role in the initiation of a robust immune response. Indeed, the yellow fever vaccine YF-17D is considered a hallmark vaccine and elicits a very potent immune response due to strong activation of both myeloid (cDC) and plasmacytoid (pDC) dendritic cells [98,99]. Interestingly, the Bartha K61 vaccine also hyperactivates the pDC population [83].

For different alphaherpesviruses, including HSV-1 and HSV-2, but also, e.g., EHV-1, there is still no efficacious vaccine. In the past, numerous HSV vaccine candidates have been developed, reaching several stages of clinical trials. Despite these endeavors, no HSV vaccine has been licensed thus far [100,101]. Often, HSV vaccine candidates disappoint in clinical trials as they do not generate satisfactory protection levels. An important hurdle is the lack of a suitable animal model that behaves as a natural HSV-1/2 host. As herpesviruses have co-evolved with their natural hosts for millennia, there is a delicate balance between immunological stimulation and inhibition. Therefore, certain processes observed in specific non-natural host species may not be applicable in the natural host and vice versa. For PRV, however, the natural host is the pig, allowing the investigation of immunological interactions under natural circumstances. Consequently, it might be an interesting approach to learn from successful alphaherpesvirus vaccines developed in the past, such as the Bartha K61 strain, to design or improve other alphaherpesvirus vaccines in the future.
